# Confidant Relations in Italy

**DOI:** 10.5964/ejop.v11i1.741

**Published:** 2015-02-27

**Authors:** Jenny Isaacs, Francesca Soglian, Edward Hoffman

**Affiliations:** a Department of Psychology, Yeshiva University, New York, NY, USA; b MIB School of Management, University of Trieste, Trieste, Italy; Academy of Special Education, Warsaw, Poland

**Keywords:** confidant, social support, social relations, Italian culture, marital relations, family, disclosure

## Abstract

Confidants are often described as the individuals with whom we choose to disclose personal, intimate matters. The presence of a confidant is associated with both mental and physical health benefits. In this study, 135 Italian adults responded to a structured questionnaire that asked if they had a confidant, and if so, to describe various features of the relationship. The vast majority of participants (91%) reported the presence of a confidant and regarded this relationship as personally important, high in mutuality and trust, and involving minimal lying. Confidants were significantly more likely to be of the opposite sex. Participants overall were significantly more likely to choose a spouse or other family member as their confidant, rather than someone outside of the family network. Familial confidants were generally seen as closer, and of greater value, than non-familial confidants. These findings are discussed within the context of Italian culture.

There is a long history of studying close relationships in humans. One of the most distinguishing features of a close relationship is a high level of self-disclosure (Parks & Floyd, 1996). The term confidant is often used to describe the select individual(s) whom one trusts in sharing important personal matters. Indeed, self-disclosure is the key feature of a confidant relationship. Over the past three decades, behavioral research has verified a measurable link between the presence of a confidant and individual wellness. The range of studies has been wide: from drug abuse and depression among American and Canadian teenagers (Nomaguchi, 2008) to condom use among young Mexican men (MacLean, 2005). Indeed, a large body of empirical work has supported the notion that, starting at an early age, there are a plethora of negative psychological, behavioral, and social outcomes associated with friendlessness or poor quality friends (Bagwell, Schmidt, Newcomb, & Bukowski, 2001; Hartup, 1996; Hartup & Stevens, 1999; Ladd & Troop-Gordon, 2003; Salmivalli & Isaacs, 2005).

Research has demonstrated that people who report having a confidant have better overall health, and are less likely to suffer from a variety of chronic medical problems, such as cardiovascular impairment (Dickens, 2004), hypertension (Holt-Lunstad, Jones, & Birmingham 2008; Thomas, 1997; Thomas, Smucker, & Droppelman, 1998), and asthma (Wainwright, Surtees, Wareham, & Harrison, 2007; see Cohen, 2004 for a comprehensive view). The lack of a confidant is also associated with greater risk for addiction (Farrell, Barnes, & Banerjee, 1995), both anxiety and depression (Newton et al., 2008; Vinokur & Van Ryn, 1993), deliberate self-harm (Webb, 2002) and suicide (Leverich, Perez, Luckenbaugh, & Post 2002), and puts individuals at a disadvantage when trying to cope with life stressors (Brown, 1980). In an investigation of British obesity and functional health, researchers estimated that the absence of a confidant reduced the functional capacity of women by five years and four years for men (Surtees, Wainwright, & Khaw, 2004).

In one of the few large cross-cultural studies published on confidants, von dem Knesebeck and Geyer (2007) investigated emotional support and health in 22 European countries. Although a definite majority of individuals in every country reported having a confidant, the percentages varied considerably—from only 77% in Italy to over 95% in Germany, Norway, and Switzerland. Across the Atlantic Ocean in Canada, Turner (1994) found that 90% of individuals reported having a confidant. This statistic was strikingly similar to what von dem Knesebeck and Geyer calculated for those living in the UK, whose ancestors settled Canada more than two centuries ago. Similarly, we find that in countries as distinct as Brazil and Taiwan, the rates of men and women indicating they have a confidant ranges from 82% to over 98% (Hoffman, Nishimura, Isaacs, & Kaneshiro, 2013; Hoffman, Nishimura, Resende, & Isaacs, 2012).

The current study focuses on confidants among Italian adults. The mere presence of a confidant has been associated with better health outcomes among Italians (von dem Knesebeck & Geyer, 2007). However, further identifying features of the confidant relationship in Italy may shed greater light on the nature of this special relationship.

## Gender and the Confidant Relationship

Results from a recent study of confidant relations among Brazilians (Hoffman et al., 2012) indicated that women were more likely than men to report having a current confidant, with both sexes being significantly more likely to have a female rather than a male as their primary confidant. It is not surprising that more women than men had a confidant and that females were the dominant sex of the confidants identified. These sex-related findings are consistent with developmental research from three separate but interfacing domains: 1) As early as preschool age, girls engage in more active help-seeking behavior than boys, and that help-seeking is a vital feature of maintaining a confidant bond (Benenson & Koulnazarian, 2008; Garland & Zigler, 1994); 2) By middle childhood if not earlier, girls demonstrate greater empathy than do boys, and that empathy is a key aspect of this close relationship (Baron-Cohen & Wheelwright, 2004; Vaswani, 2011); and, 3) By late childhood, if not earlier, girls exhibit greater self-disclosure than do boys, and that self-disclosure is another salient feature of the confidant relationship (Dindia & Allen, 1992; Vangelisti & Caughlin, 1997). Also, adulthood relationships between males tend to be less intimate than those between females (see Reis, Senchak, & Solomon, 1985). Further cross-cultural evidence can be drawn from the tendency of male, Norwegian soldiers to prefer to confide in their female colleagues than their male counterparts, even though the military is a highly gendered institution (Gustavsen, 2013), and from the role of West African women traders as communal confidant and advisor (Lo, 2013).

Italian culture has been described as one of the more "masculine" societies in the world, characterized by strong expectations of gender differences, with females being deemed as the more nurturing of the two sexes (Hofstede, 1983). Thus, the discrepancy between males and females regarding the presence of a confidant, as well as the preference for female confidants, may be particularity salient among Italians.

## Relationship Features and Positive Social Outcomes

There is evidence that it is honesty, rather than the sheer amount or depth of disclosure, that is predictive of friendship quality (Brewer, Abell, & Lyons, 2013). Across the lifespan, interpersonal trust is also regarded as a critical facet of adults’ and children’s social relationships (Rotenberg, 1991; Rotter, 1980). The formation and maintenance of interpersonal relationships is thought to largely depend on the ability to trust the partner in a relationship. Trust has been identified as one of the strongest predictors of friendship quality among children (Jones, 1991) and both marital satisfaction and longevity among adults (Kurdek, 2002). A lack of trust in close relationships with friends and family is associated with social anxiety and depressive symptomatology (Starr & Davila, 2008), as well as low self-esteem (Wissink, Deković, & Meijer, 2009). The importance of close relationships may also play a central role in the positive outcomes associated with them. For example, the rather robust relation between close friendship and happiness is largely mediated by the notion that we are important to our friend (Demir, Özen, Doğan, Bilyk, & Tyrell, 2011). In addition, proximity is another key feature that helps aid the formation and maintenance of relationships (Preciado, Snijders, Burk, Stattin, & Kerr, 2012) and impacts the frequency of interpersonal exchanges (Latané, Liu, Nowak, Bonevento, & Zheng, 1995; Verbrugge, 1983). Finally, the frequency of contact in friendships is predictive of more social trust, less stress, better health, and an increased chance of receiving help from a friend (van der Horst & Coffé, 2012). It may be important to explore relationship features such as trust, honesty, importance of the relationship, proximity, and frequency of contact, specifically within the context of the confidant relationship, in view of the positive outcomes associated them.

## Confidants and the Italian Family

Italian families are often quite intimate. During adolescence Italian children tend to maintain more frequent and longer contact with parents and siblings, and less contact with friends, compared to adolescents in other western countries (Claes, 1998). In addition, compared to other adolescents, they are more likely to confide in members of their nuclear family about intimate matters and to name a parent (rather than a friend or romantic partner) as the person with whom they felt closest (Claes, 1998). This finding suggests that even during the period of life thought to represent a period of transition away from the family and towards peers, the family continues to occupy a central, intimate role in the lives of many Italians.

There is evidence that this pattern is often perpetuated through early adulthood. Many young adult children continue to live with their nuclear family for a protracted period due to costly and extended undergraduate study, limited vocational training opportunities, under and unemployment, and cultural customs that disproportionately affect young adults in Italy (Cook & Furstenberg, 2002). These factors may induce a further strengthening of intimate ties among many Italian families and extend well into adulthood. In addition, when adult children in Italy do leave the home, it is often for the purpose of marriage, thereby transitioning from the family of origin to the marital family (see Lanz & Tagliabue, 2007). Italian adults may therefore be more likely to choose a family member (including a spouse) as their primary confidant, and regard this bond as more intimate than if involving a non-familial confidant, due to the dynamics of Italian familial life.

## The Current Study

The focus of this study was to examine features of the confidant relationship among Italian adults. Given the previous literature examining confidant relations and gender differences in intimate relationships, it is hypothesized that a vast majority of participants will have a confidant but females will be more likely than males to have a confidant. Furthermore, both males and females will be more likely to have a female confidant than a male confidant. Based on the previous literature examining the central features of many close relationships, it is also hypothesized that confidant relationships will be seen as important and will evidence a high level of trust, honesty, mutuality, and frequent contact. Furthermore, due to the intimate nature of familial relations in Italy, it is hypothesized that participants will be more likely to choose a family member as their confidant, compared to someone outside of the family. In addition, participants who choose a family member as a confidant will be closer to that person and value that person more compared to those who choose a confidant outside of the family.

Much of the current literature focuses almost exclusively on the correlates of having a confidant relationship and fails to uncover exactly who these confidants are. Unlike other areas of study (e.g., childhood friendships, romantic relationships), the area of adult confidant relationships has not benefited from a history of rich descriptive analysis. Furthermore, given that the nature of interpersonal relationships can be quite culturally imbedded, results from this study may help expand upon the meager literature that examines this intimate relationship from a specific cultural lens.

## Method

### Participants and Procedure

A total of 135 adults completed a structured questionnaire (79 females, 56 males), either electronically or on paper (95 and 40 participants, respectively). Slightly over half of the participants (*N* = 70) indicated they we currently married. A vast majority of the participants resided in northern Italy (132) and only three were from southern Italy. Their overall mean age was 41.97 years old (ranging from 19 to 75). Participants were recruited electronically or in person by the second author.

All participants were informed of the voluntary nature and purpose of the questionnaire prior to being completing it. The participants were kept anonymous and the questionnaire took approximately 10 to 20 minutes to complete.

### Measures

The survey administered was adapted from prior work examining confidant relationships (Hoffman et al., 2012; Hoffman et al., 2013). Due to the fact that this was the first Italian sample to ever complete this survey, it was translated from English to Italian and then back translated independently by two persons proficient in both English and Italian. The questionnaire opened with the following question, "Do you have someone now in your life with whom you share important personal matters? If your answer was *no*, thank you for your participation. If you have more than one, think of the person you consider your *closest* or *best* confidant and answer these questions."

Those who reported having a confidant then proceeded to answer a series of questions. Included in this questionnaire were questions about the participant's sex and age, the confidant’s sex and age, if he or she was a relative (and if so, the nature of the relation), if he or she lived within 15 minutes by car, and if the confidant also reciprocally shared personal information. Participants were also asked when was the last time they spoke with their confidant and responses were coded as "today," "not today but within the week," and "more than a week ago."

Participants were asked to rate how important the relationship was using a three-point scale ranging from one (“not very important”) to three (“very important”). In addition, they indicated how much they trusted their confidant using a three point scale ranging from one ("50%") to three ("100%")". Finally, they reported on how often they lied to their confidant on a four-point scale ranging from one (“never”) to four (“often").

## Results

As anticipated, participants overwhelmingly reported the presence of a confidant (91.9%). Although the results were marginal, Italian women (94.9%) were more likely than men (85.7%) to have a confidant, *χ*^2^(1, *N* = 135) = 3.44, *p* = .06. Table 1 displays the breakdown of confidant sex, based on the sex of the participant. As hypothesized, Italian men were significantly more likely to have a female confidant rather than a male confidant, *χ*^2^(1, *N* = 48) = 21.33, *p* <.001. In contrast, females were significantly more likely to have a male confidant rather than a female confidant, *χ*^2^(1, *N* = 75) = 14.52, *p* < .001. The cross-sex preference may be accounted for by married individuals choosing their opposite-sex spouses. Indeed, married males and females did show a significant cross-sex preference, *χ*^2^(1, *N* = 27) = 23.15, *p* < .001; *χ*^2^(1, *N* = 39) = 18.69, *p* < .001; respectively. Single males and females did not show this preference, *χ*^2^(1, *N* = 21) = 2.33, *p* = .13; *χ*^2^(1, *N* = 36) = 1.00, *p* = .32; respectively. In addition, married individuals were significantly more likely to choose their spouse (70.1%) than someone who was not their spouse (29.9%); *χ*^2^(1, *N* = 77) = 12.48, *p* < .001.

**Table 1:** Breakdown of Confidant Sex Based on Marital Status and Sex of the Participant

Sex of Participants	Sex of Confidant	
	% males	% females
All		
Men	16.7	83.3
Women	72.0	28.0
Single		
Men	33.3	66.7
Women	58.3	41.7
Married		
Men	3.7	96.3
Women	84.6	15.4

Overall, the confidant relationship tended to have many positive features. It was generally viewed as personally important, high in mutuality and trust, and involving minimal lying. In fact, 96% of participants indicated that the confidant relationship was reciprocal. In addition, most individuals (91.9%) rated the relationship as "very important" to them, 8.1% rated it as "somewhat important", and no one rated it as "not very important", *χ*^2^(1, *N* = 123) = 86.3, *p* < .001. A substantial majority (78.9%) said they trusted their confidant 100%, 18.7% said they trusted them 75%, and only 2.4% said they trusted their confidant just 50%, *χ*^2^(2, *N* = 123) = 119.6, *p* < .001. Finally, participants were significantly more likely to "never" or "rarely" lie to their confidant (90.2%), compared to "sometimes" or "often" lie (9.8%), *χ*^2^(1, *N* = 123) = 79.6, *p* < .001.

Confidants also tended to speak fairly frequently to one another. Three quarters of individuals (74.4%) indicated they spoke with their confidant the same day as the survey, 24.5% had not talked today but had talked within the last week, and only 4.1% indicated that they had last spoken to their confidant more than a week ago, *χ*^2^(2, *N* = 99) = 70.12, *p* < .001. Individuals who had spoken to the confidants the same day were significantly more likely to believe their confidant relationship was reciprocal, *χ*^2^(2, *N* = 98) = 22.80, *p* < .001; important, *χ*^2^(2, *N* = 98) = 11.62, *p* < .01; and based on 100% trust, *χ*^2^(4, *N* = 98) = 22.63, *p* < .001. The reverse pattern was also evident for individuals who had not spoken to their confidants for over a week. Those who indicated that they had not had contact with their confidant for over a week disproportionally reported often lying to them, *χ*^2^(6, *N* = 99) = 14.09, *p* < .05. Overall, this suggests that very frequent contact with a confidant is associated with more positive relationship features.

Significantly more people choose spouses or other family members (64.2%), compared to individuals outside of the family (35.8%), as their confidants, *χ*^2^(1, *N* = 135) = 9.96, *p* < .01. Nearly 70% of familial selections were spouses, with relatively few choosing either a parent (10.4%) or a child (5.2%). Compared to those who selected non-familial confidants, individuals who chose family members tended to be older, *t*(121) = 2.45, *p* < .05, and to have an older confidant, *t*(119) = 2.58, *p* < .05. Those who chose familial confidants were also more likely to be married than single, with the reverse being true for those who had a non-familial confidant, *χ* ^2^(1, *N* = 128) = 6.22, *p* < .05. Understandably, participants with familial confidants were more likely to live less than 15 minutes away by car from them, *χ* ^2^(1, *N* = 123) = 40.83, *p* < .001, more likely to have spoken to their confidant on the day of the survey, and less likely to have last spoken to them later on in the week or more than a week ago, *χ* ^2^(1, *N* = 98) = 15.62, *p* < .001. In addition to these potential markers of closeness, confidants who were family members were valued as significantly more important than confidants outside of the family, *χ* ^2^(1, *N* = 123) = 91.87, *p* < .01, and were trusted more, *χ* ^2^(2, *N* = 121) = 119.61, *p* < .01. See Table 2 for comparisons of participants who did or did not choose a family member as their confidant.

**Table 2:** Characteristics of Participants who Choose a Familial or Non-Familial Confidant

Characteristic	Familial Confidant	Non-Familial Confidant
Marital Status^a^		
Single	38.7%	62.4%
Married	58.1%	32.3%
Distance Apart by Car		
≤ 15 Minutes	64.5%	26.1%
> 15 Minutes	35.5%	73.9%
Importance of Relationship		
Not Very	0.0%	5.5%
Some-what	13.3%	45.1%
Very	88.7%	49.5%
Last Time Spoke		
Today	86.0%	51.2%
This Week	14.0%	39.0%
> a Week ago	0.0%	9.8%
Level of Trust in Confidant		
50%	2.5%	2.3%
75%	10.1%	34.1%
100%	87.3%	63.6%

^a^Percentages do not add to 100 percent because approximately 5% of participants chose a marital status other than Married or Single.

## Discussion

In this study, a vast majority of Italian adults (91%) identified a current confidant in their lives. As this percentage was higher than the number (77%) reported by von dem Knesebeck and Geyer (2007), it may reflect the fact that our investigation could have included a smaller percentage of elderly participants; this is a demographic at greater risk of lacking a confidant, partially due to death among the similar age individuals, especially spouses, that are often selected as confidants (Sijuwade, 1994).

### Confidant Characteristics

It is clear that the quality of confidant relations in Italy was highly positive. Participants generally seemed to maintain frequent verbal contact and overwhelmingly described the relationship as both reciprocal and personally important. They also reported high levels of trust and limited amounts of lying. Such positive features are particularly noteworthy because relationship quality has been found to be a key factor in determining positive outcomes from a social relationship (Bradbury, Fincham, & Beach, 2000; Halford, Kelly, & Markman, 1997; Walker, Hart, & LaJoie, 2007). For example, low quality, conflict-ridden relationships may actually have deleterious psychological effects on its constituents (Hawkins & Booth, 2005).

Not unexpectedly in the Italian social context, we found that familial confidants were regarded as significantly closer emotionally than confidants from outside the family. This finding may reflect the strong ties that often still exist within the modern Italian family (Sgritta, 1988). Even as marital instability becomes more pervasive in many areas of Europe, Italian marriages continue to be relatively more resilient (Vignoli & Ferro, 2009). However, in light of the strong parent- child bonds extant in Italian culture (Reher, 1998), it was somewhat surprising that neither parents nor adult children were often selected by our sample as the primary confidant. One explanation may be that a majority of participants were over 40 years old and had likely moved out of their parents’ homes. Quite possibly, too, Italian parental ability to withhold valuable emotional and financial resources from their adult children (Rosina & Fraboni, 2004) may discourage their disclosure of emotionally sensitive material, in order to avoid parental disapproval. Furthermore, concerning the older participants, the added possibility exists that one or both parents were deceased. The fact that a majority of our sample were married and past the phase of young adulthood might have led to having more opportunities to view their grown child as a confidant, but this situation was rare. Perhaps it would have been more pronounced in a more elderly sample, especially given that adult children are often chosen as confidants among widows (Wenger & Jerrome, 1999).

As we predicted, females were somewhat more likely than males to report a confidant, and males were more likely to choose a female. However, females were not more likely to choose other females as their primary confidant. This finding seems to be partially due to the tendency of individuals to identify their spouse as primary confidant. Female preference for female confidants might be more salient during either early adulthood (prior to marriage), or old age, when husbands are more likely to have significant mental or physical impairments or be deceased. Consistent with their counterparts in Wales (Wenger & Jerrome, 1999), married individuals in Italy are far more likely to choose their spouse as their confidant. This finding is understandable given that self-disclosure to one’s spouse is a key feature of a successful marriage (Clark & Lemay, 2010; Hendrick, 1981).

### Family in Italian Culture

Although there has been a shift in the centrality of marriage in many countries over the last few decades, this shift may not be as pronounced in Italy as it is in many other European countries (Luciano et al., 2012; Rosina & Fraboni, 2004). For example, Rosina and Fraboni argued that although the age of marriage is shifting upward in Italy, its crucial role as a major life transition is nevertheless still apparent among Italians. For instance, the act of permanently leaving one’s parental home is more frequently associated with marriage in Italy than in many other Western countries. The role of family, in general, may be particularly important in the lives of Italians. As reviewed in Rosina and Fraboni, not only do parents maintain strong emotional ties even after their children enter adulthood, they also often maintain both financial and residential closeness to their adult children. For example, it is not uncommon for parents to financial assist their child (e.g., purchase their first house) and often family members continue to live in close proximity to one another, even in adulthood. Close familial relationships, coupled with the continued belief that marriage is a fundamental value, may explain why the Italians in this study were more likely individuals from other countries, such as Taiwan and Brazil (Hoffman et al., 2012; Hoffman et al., 2013), to select a family member as their primary confidant.

### Limitations and Future Studies

The present study captured only a single snapshot in the lives of Italian adults. Future studies would benefit from following the role of the confidant relationship among Italians over their lifespan. The changing dynamics of relationships may be especially salient in contemporary Italy, where many people are making a slow transition away from a traditional family structure (Luciano et al., 2012; Rosina & Fraboni, 2004). Previous longitudinal studies, such as the Bangor (Wales) Longitudinal Study of Ageing (Wenger & Jerrome, 1999), have revealed interesting developmental changes that occurred in confident relations among the elderly. Moreover, a more detailed exploration among different age groups might yield a more developmentally sensitive understanding of the confidant relationship among Italians.

Another possible limitation is the primarily descriptive nature of the current study. Nevertheless, results from this study have the potential to advance the field in describing this valuable relationship. In light of the paucity of empirical information on the characteristics of confidants, we hope that this study will help serve as a springboard for more complex evaluations regarding the nature of this relationship and encourage exploration that is couched within varying cultural contexts. Future studies may also benefit from utilizing more complex measurements that provide a greater range of coverage by including multi-item scales.

Despite these limitations, the current study helps to shed light on the confidant relationship among adults in Italy. Our findings reinforce the conception that its constituents regard their bond as both positive and important, and also affirm the centrality of the family in Italian social life today. In contrast to cultures like China’s, in which emotional intimacy in marriage has traditionally taken a backseat to e*nqing,* the practice of expressing gratitude and admiration between spouses (Chen & Li, 2007), contemporary Italy seems to have retained the value of keeping the marital partner in particular as the primary target of trusted, intimate disclosure.
